# A Force-Feedback Methodology for Teleoperated Suturing Task in Robotic-Assisted Minimally Invasive Surgery

**DOI:** 10.3390/s22207829

**Published:** 2022-10-14

**Authors:** Armin Ehrampoosh, Bijan Shirinzadeh, Joshua Pinskier, Julian Smith, Randall Moshinsky, Yongmin Zhong

**Affiliations:** 1 Robotics and Mechatronics Research Laboratory (RMRL), Department of Mechanical and Aerospace Engineering, Monash University, Melbourne, VIC 3800, Australia; 2 Data61, CSIRO, Brisbane, QLD 4069, Australia; 3 Department of Surgery, Monash University, Melbourne, VIC 3800, Australia; 4 Department of Mechanical and Automotive Engineering, RMIT University, Melbourne, VIC 3083, Australia

**Keywords:** robotic assisted minimally invasive surgery, haptic feedback, force sensing, robotic needle driver, optoelectronic sensor, virtual fixture

## Abstract

With robotic-assisted minimally invasive surgery (RAMIS), patients and surgeons benefit from a reduced incision size and dexterous instruments. However, current robotic surgery platforms lack haptic feedback, which is an essential element of safe operation. Moreover, teleportation control challenges make complex surgical tasks like suturing more time-consuming than those that use manual tools. This paper presents a new force-sensing instrument that semi-automates the suturing task and facilitates teleoperated robotic manipulation. In order to generate the ideal needle insertion trajectory and pass the needle through its curvature, the end-effector mechanism has a rotating degree of freedom. Impedance control was used to provide sensory information about needle–tissue interaction forces to the operator using an indirect force estimation approach based on data-based models. The operator’s motion commands were then regulated using a hyperplanar virtual fixture (VF) designed to maintain the desired distance between the end-effector and tissue surface while avoiding unwanted contact. To construct the geometry of the VF, an optoelectronic sensor-based approach was developed. Based on the experimental investigation of the hyperplane VF methodology, improved needle–tissue interaction force, manipulation accuracy, and task completion times were demonstrated. Finally, experimental validation of the trained force estimation models and the perceived interaction forces by the user was conducted using online data, demonstrating the potential of the developed approach in improving task performance.

## 1. Introduction

In the past 20 years, minimally invasive surgery (MIS) techniques have been developed to minimize the size of incisions needed to access target organs. This technique is an alternative to open surgery, in which the surgeon has to cut open the patient’s body in order to gain adequate sight and workspace to utilize conventional instruments. In the MIS approach, the surgeon makes a few small incisions instead of one large one, making the process less invasive. As a result of the MIS approach, patients can recover more quickly, suffer less trauma, have less postoperative pain, and stay in the hospital for a shorter period of time. This results in reduced costs and burden on the healthcare system [1].

MIS is performed with manual handheld instruments or robotic systems [2,3]. The advantages of robot-assisted surgical systems over manual MIS instruments include higher dexterity and active degrees of freedom (DOF), enabling the surgeon to perform a wider range of maneuvers with enhanced precision [4,5,6]. While robotic systems can provide many benefits to surgeons and patients, they also pose significant challenges. As compared to manual instruments, the main drawback of robotic surgical systems is the degradation of force feedback, also known as haptic feedback [7,8]. A mechanically designed device in manual MIS provides the surgeon with a clear sense of force during the tool–tissue interaction. It is essential that the surgeon be aware of this information, which is left out in robotic systems where the surgeon only relies on visual cues of the interaction between tool and tissue. Additionally, due to the complexity of operation of robotic instruments, high dexterity comes at the cost of longer operation times [9]. Consequently, new robotic suturing methodologies are needed that improve task completion times while ensuring high force-sensing accuracy.

Suturing is an important surgical task that requires special attention due to its inherent complexity and requires effective approaches to overcome it. The high level of concentration and coordination required to perform the task in a confined space contributes to fatigue and reduces the quality of the suture. It requires highly skilled surgeons experienced with telesurgery and multi-DOF slave robots in order to complete this task because of the limited space and complex tool paths. There has been considerable interest in the development of approaches and new features for robotic systems that tackle issues associated with teleoperation [10,11,12]. They provide surgeons with tools for improving the surgical site’s perception or making surgical tasks more efficient [13]. Researchers have investigated several approaches to improve teleoperated suturing. Among these approaches are adding haptic feedback and utilizing active constraints to assist the surgeon when suturing.

The remainder of this paper is organized as follows: first, an overview of haptic feedback control methodologies is provided, including force sensing approaches and existing virtual fixture algorithms for suturing task being reviewed. The next section discusses the proposed force sensing instrument, focusing on indirect force estimation using data-based models, and haptic feedback impedance controller design. Next, a VF algorithm is developed utilizing geometry construction and hyperplanar architecture. The geometry construction process includes calibrating reflective optoelectronic sensors, generating point clouds, and estimating tissue planes. A description of the experimental research facility and the hardware setup follows. The Results section describes the details of the experiments conducted and the results obtained to validate force models and characterize the effectiveness of the VF algorithm. Finally, the conclusion and discussion of the results conclude the paper.

## 2. Overview of the Robotic Teleoperation Surgery Approaches

To minimize damage to the tissue during suturing, it is necessary to perceive the force exerted by the tool on the tissue. The lack of force feedback to surgeons has been reported as the main limitation of current RAMIS systems [14], contributing to increased injuries during operations [15], suture breakage, and tissue damage [16]. Furthermore, regulating the operator’s input motion commands has also been shown to be an effective method for assisting the surgeon during complicated robotic surgery procedures. In a similar way to mechanical fixtures that limit surgical tool motion, virtual fixtures achieve this goal in a more flexible and adaptive manner.

### 2.1. Haptic Feedback and Force Sensing

Through a bilateral control architecture, haptic feedback transmits force data from the robot–patient interactions to the surgeon side to provide a real-time interactive environment between the surgeon and the surgical field [17,18,19]. A number of studies examined the effect of haptic feedback on suturing accuracy and performance. Tavakoli et al. [20] showed that providing haptic feedback for the stitching task is a trade-off between the task completion time and the magnitude of the applied forces to the tissue. In contrast, a more recent study by Talasaz et al. [21] demonstrated that force feedback improved both maximum applied force on the tissue and completion time for the stitching task. A study conducted by Currie et al. [9] compared the efficiency of visual feedback and direct force feedback on reducing maximal forces through customized Quanser haptic wands, concluding that direct force feedback reduces maximum forces more effectively than visual feedback. A haptic interface customized to the suturing task was used by Carreras et al. [22] to investigate the influence of direct force and torque feedback on the accuracy of suturing tasks in a virtual reality environment.

Developing direct force feedback requires supplying force data from the tool–tissue interaction to the surgeon. An in-depth overview of force sensing methods and state of the art of haptic feedback for MIS applications were presented in recent review papers [23,24]. Various approaches to force sensing have been proposed in the literature, including sensor-based and sensor-less force measurements [25,26].

Sensor-based force measurement techniques include using capacitive, piezoelectric, piezoresistive, optical sensors, etc. [27]. It is ideal to locate the force sensor near the tip of the instrument [26]. Kuebler et al. [28] proposed an integrated sensing unit inside the end-effector, capable of 6-DOF force and torque measurement. Using shape deposition manufacturing, Dollar et al. [29] proposed embedding a strain gauge in the end-effector. The development of capacitive transducers and fiber optic force sensors has also been reported [30,31]. However, the sterilization process is still a challenge for this approach that uses a harsh procedure to kill bacteria with heated steam or chemical sterilization. It is therefore necessary for the sensors to be biocompatible and sterilizable. Moreover, the sensing unit adds significant cost to disposable instruments and instruments measuring force in multiple DOF. Although recent advancements have been made, force sensing with this approach requires further downsizing due to the fact that many surgical instruments have millimeter-scale end-effectors.

Several approaches have been proposed in the literature to avoid such issues. Contact-less methods use sources such as image data, lasers, and optical coherence tomography for force-sensing [32,33]. Marban et al. [34] developed a vision-based approach to force estimation using a convolutional neural network and a long-short term memory network. There are, however, limitations to this method due to factors such as lighting conditions. Another solution is force estimation, which utilizes information such as known robot dynamics, motor current, and encoder data to eliminate dealing with force sensors [35]. However, this approach needs to be improved in terms of accuracy when compared to sensor-based approaches. Installing the force sensing unit away from the instrument tip is another alternative solution to overcome the challenges of placing the sensors at the tip [36]. An instrument capable of measuring interaction forces with force sensors proximally located at the top of the tool has been developed in [37]. However, the force-sensing methodologies still require further improvement in terms of measurement accuracy, sterilization, and cost. There are also many studies on force-sensing technology development which lack accuracy estimation and bench-marking for future comparison, among other approaches [24].

### 2.2. Virtual Fixture

Virtual fixtures (VF), also known as active constraints, are software-imposed enforcements that regulate the user’s motion and provide abstract sensory information in addition to the other sensory feedback from the remote environment [38]. When VF is implemented, a collaborative control strategy is used, in which the tool movement is monitored by the robot controller while the human user controls the robotic arm. The controller detects any deviation from the planned trajectory or entering a restricted region. Then, the controller imposes an active force on the user through the master device to guide the user back to the planned trajectory or nullifies the user command to enter a restricted region.

There are several classifications for VFs based on their properties. According to one classification, the constraints are divided into two groups: regional constraint and guidance constraint [39,40]. Regional constraints, also referred to as forbidden region VFs, restrict slave manipulators’ movement to the desired region specified by the surgeon during the intraoperative planning process. A haptic system utilizing forbidden region virtual fixtures will increase stiffness felt by the user when entering the undesired zone to prevent damage to the tissue [41]. Several benefits can be gained from using this type of VF, such as avoiding damage to the protected organs and regions [42], preventing kinematic singularities [43], and simplifying tasks [44].

There have been several studies that have investigated the use of virtual fixtures for suturing. An impedance active constraint was proposed by Chen et al. [45] to develop virtual fixtures that assist with stitching and knot tying. Experiments showed that VF improved needle exit point accuracy, task completion time, and overall user workload. Using a telemanipulation system, Fontanelli et al. [46] compare a number of control strategies to assist the operator during stitching tasks. A guidance virtual fixture was implemented to constrain the tool’s position along the desired trajectory as part of the shared control strategy. Selvaggio et al. [47] investigated the issue of needle re-grasping during suture task. A new haptic-guided control method was developed to enable the user to grasp the needle more effectively while avoiding the limitations and singularities of robot joints. An online optimization trajectory generation approach was developed by Colan et al. [48] for implementing active constraints during endonasal surgery stitching. In order to prevent damaging surrounding nasal tissues, they used sequential convex optimization for online needle trajectory generation. In spite of advances in virtual fixture methods to facilitate suturing task development, performance enhancement has remained limited and requires further progress. It is mainly due to the inherent complexity of telerobotic suturing, such as the high cognitive demands placed on the surgeon when controlling all of the required DOFs when performing suturing.

In this paper, a new force-sensing and semi-automated robotic needle driver concept design is presented for facilitating teleoperated MIS suturing tasks. Through haptic feedback control architecture, the proposed methodology provides the surgeon with two sets of force information: virtual fixture force and needle–tissue interaction force. The VF algorithm prevents needle–tissue accidental contacts and maintains ideal end-effector-tissue distance. Additionally, the proposed end-effector mechanism at the tip of the force sensing tool generates the desired trajectory using a rotating DOF that limits needle movement along its curvature. The authors believe that this research is one of the first attempts to study a semi-automated needle driver with virtual fixture assistance to simplify the suturing task. Furthermore, to enhance sensory perception and improve task safety, a data-based and indirect force estimation model was utilized to establish a direct force feedback architecture. To the best of our knowledge, this is the first study to investigate the estimation accuracy of a hybrid approach combining proximal sensing with force estimation techniques. The main contributions of this research are as follows:

- Development and characterization of a force-sensing needle driver with a proof-of-concept end-effector;
- Investigating data-driven models for indirect needle–tissue interaction force estimation using the proposed force sensing tool and validating the model with experimental results;
- Establishing a hyperplanar virtual fixture to facilitate teleoperated suturing using a new reflective optoelectronic sensor-based approach.

## 3. Force Sensing Semi-Automated Robotic Needle Driver

The investigated teleoperated robotic system consists of two main subsystems, the user interface and Phantom master device at the surgeon’s side and the slave robot at the patient’s side. The developed MIS robotic needle driver comprises a cable-driven end-effector for semi-automated suturing with a force sensing instrument for measuring force during needle–tissue contact. In order to approximate the needle–tissue interaction forces, the force sensing instrument employs an indirect force measurement approach. A data-driven force model was developed using the measured forces from the tool’s force sensor and the needle insertion motor’s rotational position. This force model created a mapping between robot sensor data and needle–tissue interaction forces. Finally, the interaction forces were conveyed to the master device to enhance sensory perception, thus improving the quality of the suturing process and minimizing tissue damage.

### 3.1. Force Sensing Instrument

**Figure 1:**
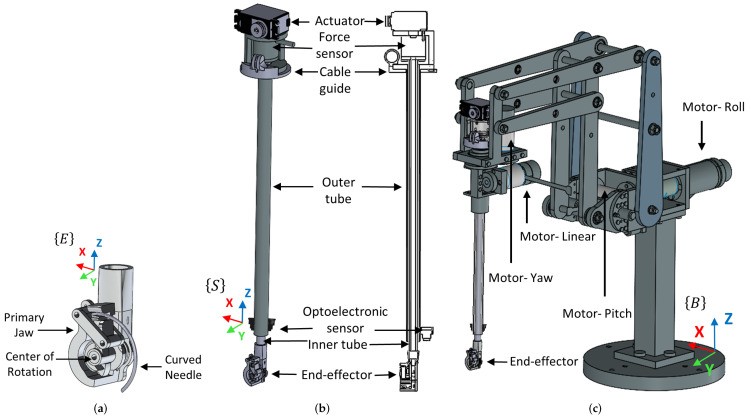
Proposed robotic suturing system. (**a**) end-effector; (**b**) force-sensing semi-automated needle driver instrument; (**c**) MIS robotic arm.

In the following section, various components of the slave robot on the patient’s side will be discussed. The force-sensing needle driver consists of a force/torque (F/T) sensor, tool shafts, the end-effector, and an actuator that drives the end-effector, as shown in Figure 1.

The cable-driven end-effector mechanism has previously been presented in our research project [49]. This end-effector was designed to reduce workload and simplify the suturing task, enabling it to be integrated into an automated system. The mechanism’s working principle is based on decoupling the required needle insertion movements into only one rotating DOF utilising a primary jaw responsible for applying force to the end of the needle around a fixed centre of rotation (Figure 1a). This mechanism was designed to pass the needle through its curvature.

The end-effector is integrated into the inner shaft of a force-sensing surgical instrument, and the F/T sensor is located at the proximal end, as shown in Figure 1b. As shown in this figure, the F/T sensor was enclosed in a casing to prevent contact with any other components besides the inner tube. In order to protect the F/T sensing unit from external forces such as tool–incision interaction or frictional loads, the inner shaft passes through an outer tube. This resulted in an isolated measurement of tool–tissue interaction. Furthermore, a cable guide was mounted under the F/T sensor to prevent contact between the driving cables and the sensor. Because of the inner tube and end-effector weight components, gravity affects the measurement of the proximal force sensor. In order to account for gravitational forces, a bias was applied to the force sensor just before recording the force data for each needle insertion cycle in order to remove the influence of tool weight on force measurements. A reflective optoelectronic sensor was installed at the tip of the instrument’s outer tube. The sensor was rigidly glued to the outer tube at a specific distance from the tip. The optoelectronic sensor was used to measure the distance between the end-effector and the tissue surface using infrared.

Finally, the force sensing instrument is integrated into a 4-DOF robotic arm equipped with actuators that rotate the instrument in roll, pitch, and yaw, as well as linearly along the instrument shaft, as shown in Figure 1c. It features a double parallelogram structure with a remote centre of motion (RCM) mechanism to avoid mechanical contact between the robot and the incision point during motion.

### 3.2. Data-Based Interaction Force Estimation Using Neural Network Models

The indirect needle–tissue interaction force estimation model creates a mapping from the robotic instrument sensor data, including tool force sensor data and needle insertion motor kinematics, to the actual needle–tissue interaction forces. Data-based models were constructed using neural network architecture to estimate the interaction forces. The neural network model has been shown to be capable of modeling any nonlinear input–output relationship when the network size and training are sufficient. There are three layers in a neural network: the input layer, the hidden layer, and the output layer. The number of hidden layers varies depending on the required accuracy and the network size to approximate the desired function. Hidden layer output is expressed as follows:

(1) $$O_{h}=s\left(W_{h}u+b_{h}\right)$$

where *s* is a sigmoid function, and $O_{h}$ is the vector of hidden layer outputs. $W_{h}$ and $b_{h}$ are the hidden layer weights matrix and bias coefficients vector, respectively. The number of time steps in each input vector is another parameter that needs to be adjusted in the training phase. The output layer calculates the estimated force values in each time-step $\hat{y}$ from the hidden layer output values $\left(O_{h}\right)$ using the following equation:

(2) $$\hat{y}=l\left(W_{o}O_{h}+b_{o}\right)$$

where *l*, $W_{o}$ and $b_{o}$ are a linear function, the output layer weights matrix and bias coefficients vector, respectively. The weight matrices and bias vectors were calculated using the Levenberg–Marquardt (LM) algorithm. This algorithm is a standard gradient descent method for training neural networks based on a dataset containing the input and target output values.

Force estimation was performed using a recurrent neural network (RNN) architecture. The RNN structure had one external output to input feedback connection. Therefore, the input vector included estimated force values feedback to the system from the output. This form of network is known as nonlinear autoregressive with external input (NARX). The relationship between the input vector u and the estimated force $\hat{y}$ at each time-step *t* can be written as follows:

(3) $$\hat{y}\left(t\right)=f\left(u\left(t-1\right),\ldots ,u\left(t-d\right),\hat{y}\left(t-1\right),\ldots ,\hat{y}\left(t-d\right)\right)$$

where *f* is a nonlinear function resulting from substituting Equations (1) into (2) and *d* is the number of subsequent time steps of input vector. The input vector *u* consists of three measured force components of the tool–force sensor $\left(f_{x},f_{y},f_{z}\right)$ and motor position and velocity $\left(\theta ,\dot{\theta }\right)$ in each time-step. The dependent output value $\hat{y}$ is regressed on d number of previous time-steps of the input vector $\left(u\left(t-d\right)=\left(f_{x},f_{y},f_{z},\theta ,\dot{\theta }\right)\right)$, as well as output vector $\hat{y}\left(t-d\right)$.

In the suturing process, the needle and tissue come into contact in two phases, during needle insertion and needle extraction. In this study, the force models were trained for needle insertion, and the force data from needle extraction were not considered during training. The needle insertion stage begins when the needle tip touches the tissue surface, and the needle passes through and cuts it. The needle insertion phase involves active cutting forces that puncture the tissue, whereas the extraction phase does not include such forces. In the extraction phase, the forces are primarily caused by friction between the needle and the tissue as it passes through the already punctured tissue. Therefore, by providing force feedback during needle insertion, the surgeon can improve his or her sensory perception of the tissue stiffness, reducing tissue damage.

**Figure 2:**
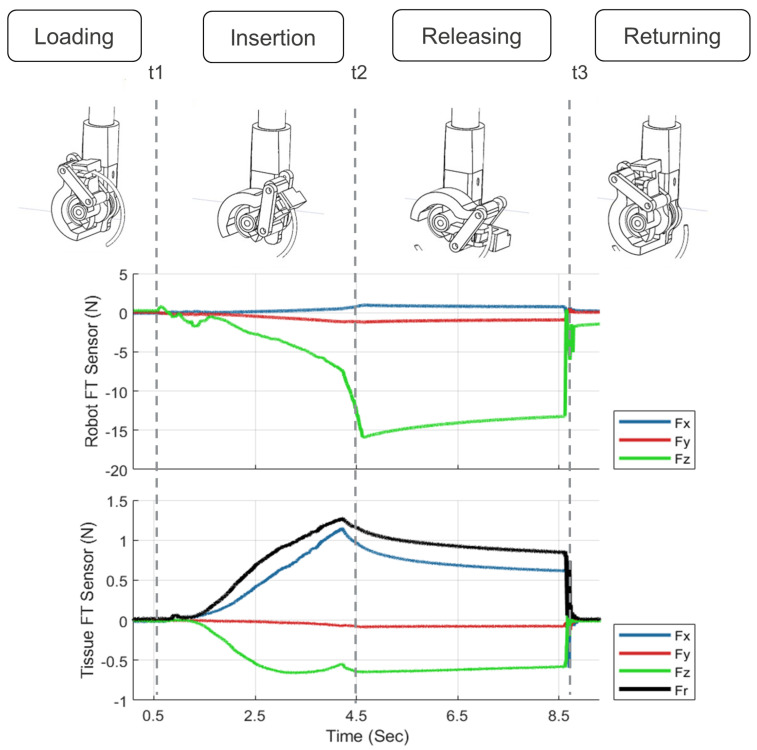
The recorded robot and tissue force sensors data for one cycle of needle insertion.

Figure 2 shows a sample of input–output pairs recorded by the force sensing instrument for one needle insertion cycle. Input data include needle insertion motor kinematics and F/T sensor data of the instrument. The target output is the needle–tissue interaction resultant force $F_{r}$ of the three Cartesian force components measured by a force sensor underneath the tissue base. The needle insertion process begins by loading the needle driver and approaching the needle entry point. The force sensors are biased at this point and have zero force component measurements (until time $t_{1}$). The measured force components by both force sensors and the motor position increase during the insertion cycle until the needle tip emerges at the exit point (until time $t_{2}$). Force estimation models were developed using $t_{1}$ and $t_{2}$ interval data. In this interval, the recorded instrument’s force sensor data contain the necessary needle driver force components applied to the needle during needle insertion to overcome tissue resistance. Following this stage, the force values and motor position were relatively constant until the jaw reached the first mechanical stopper (at time t3). After the jaws are returned to their initial position, the force values are back to zero with the exception of a small tool force sensor component, $F_{z}$, which measures jaw opening force.

Networks were trained using 8725 input–output samples, including needle insertion cycles with various rotational velocities from various silicone tissue locations. This dataset was divided into three sets of 5672, 1745, and 1308 samples for training, validation, and test dataset, respectively. In order to evaluate the model performance, the trained networks were tested against an additional unseen dataset. There was only one hidden layer used to model the dynamic relationship of the needle driver. One hidden layer would reduce computational costs and provide a suitable model for real-time applications. The number of investigated hidden nodes was from 10 to 50 nodes, increasing by increments of 5. The number of delays considered ranged from 1 to 5, increasing by one increment. To reach a model with minimal complexity and desired accuracy, the means square error (MSE) of the test dataset estimation was checked against a threshold for each trained model. Moreover, a minimum delay value is desired to minimize the lag in the force estimation model.

Following the training phase, a model with suitable estimation MSE and network size was selected. The selected RNN model consisted of 15 nodes in the hidden layer with d=2 with estimation MSE of $10^{-4}$ N for the training dataset.

### 3.3. Needle–Tissue Interaction Haptic Feedback Using Impedance Control

An impedance control approach was used to provide estimated interaction force data to the user using a phantom haptic device. As a result of the end-effector’s unique design, the needle could be rotated along its curvature independently of other DOFs of the robot. Therefore, only one DOF was required to provide the haptic feedback of needle–tissue interaction. The dynamic equation of the interaction between the haptic device and the operator for the considered DOF (x-direction) can be described as follows:

(4) $$M_{mx}{\ddot{x}}_{mx}+C_{mx}{\dot{x}}_{mx}=f_{mx}+f_{hx}$$

where $M_{mx}$ is the master robot inertia coefficient, $C_{mx}$, is the master robot damping coefficient, ${\dot{x}}_{mx}$ and ${\ddot{x}}_{mx}$ are the master device velocity and acceleration in the *x*-direction, respectively. The forces $f_{mx}$ and $f_{hx}$ are the master device control force and the user applied force in the *x*-direction, respectively.

Based on the transparency definition, teleoperated systems must have equal user interaction forces with the master device and slave arm forces on the tissue. To provide a transparent sense of environment forces $f_{e}$ from the needle–tissue interaction forces, the haptic force $f_{mx}$ was defined such that it compensates for the haptic device inertia as follows:

(5) $$f_{mx}=M_{mx}{\ddot{x}}_{mx}+C_{mx}{\dot{x}}_{mx}-\alpha_{F}{\hat{f}}_{e}$$

where ${\hat{f}}_{e}$ is the estimated needle–tissue interaction force using the force mapping model, and $\alpha_{F}$ is an environmental force scaling factor.

Phantom has no force sensors to directly measure forces, so the force applied by users to the haptic device was estimated. The transmitted environment forces to the user can be estimated as follows:

(6) $${\hat{f}}_{hx}=\alpha_{F}{\hat{f}}_{e}$$

The selection of $\alpha_{F}$ affects the system’s stability. In the following experiments, this parameter was empirically selected ($\alpha_{F}=0.5$) such that the user can easily overpower scaled environment force.

The system stability is greatly influenced by the biomechanical impedance of the user’s interaction with the haptic device, which can vary considerably [50]. When a user grasps the haptic device, they can either stabilize a previously unstable system or destabilize a previously stable system depending on whether they direct energy toward the system or dissipate it. After testing multiple scaling factors, the user selected 0.5 based on his ability to maintain the stability of the system throughout the experiments without experiencing discomfort and fatigue. However, the scaling factor could be equal to one without destabilizing the system depending on the user.

## 4. Hyperplanar Virtual Fixture Utilising a Reflective Optoelectronic Sensor

The VF algorithm aims to maintain the ideal distance between the end-effector and tissue surface ($L_{H}$) and prevent unwanted needle–tissue contact. The hyperplanar virtual fixture geometry was developed by estimating the local tissue plane based on the distance data recorded by the optoelectronic sensor. The incorporation of optoelectronic sensors into teleoperated robotic systems was found to enhance human operator input during tool fine alignment [51].

**Figure 3:**
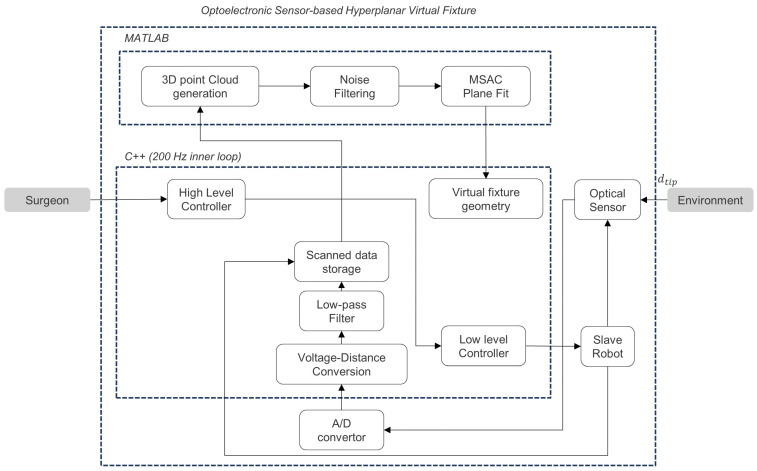
Schematic block diagram of the optoelectronic sensor-based algorithm for hyperplanar VF geometry construction.

Figure 3 illustrates the schematic block diagram of the proposed algorithm for constructing the hyperplanar VF geometry by using a reflective optoelectronic sensor or optic sensor. Following a choice of the needle entrance point expressed in the robot’s base coordinate frame, the algorithm began. The high-level controller generated a circular path for the robot to scan the tissue around the entrance point and sent the required commands to the robot motor low-level controllers to follow this path. In this step, the local coordinates for samples taken near the desired point on the tissue surface were provided. The optical sensor generated a voltage according to the distance between the tissue sample points and the robot tip. Following the analog to digital conversion of sensor voltage data, a voltage to distance mapping was performed. The collected distance data were then filtered using a 20th-order one-dimensional median filter to remove the noisy content. The code for the robot was written in C++ and ran at a rate of 200 Hz.

In the next step, Matlab was used to generate the 3D point cloud based on the distances collected by the optical sensor and corresponding robot joint coordinates. Before the plane estimation step in Matlab, 3D point cloud data were subjected to noise cancellation. Using filtered data, the plane estimation algorithm approximated the orientation of the tissue plane. The estimated tissue plane orientation was then used for defining the hyperplane parameters in VF geometry construction.

### 4.1. Reflective Optoelectronic Sensor-Based VF Geometry Construction

Reflective optoelectronic sensors work by transmitting and receiving infrared signals. Distance is calculated by comparing the properties of the sent and received waves and the time taken to receive them. An optical sensor generates an output voltage based on the sensed signals using a combination of a position-sensitive detector, an infrared emitting diode, and a signal processing circuit. The measurement is relatively robust with various reflectivity percentages or environmental conditions due to incorporating the triangle method.

Over the sensor’s usable range, there is approximately a linear relationship between the output voltage and the inverse of the distance between the sensor and the object, according to the sensor’s datasheet. Sensor calibration was performed on silicone tissue samples in order to determine the best-fit model between voltage and inverse distance. A least-squares curve fitting method was used to calculate the following model using the recorded output voltage *v* and the known distance between the optic sensor and tissue surface $d_{tip}$:

(7) $$d_{tip}=9.041e^{4}\;{\left(v\times 366.2\right)}^{-1.22}$$

**Figure 4:**
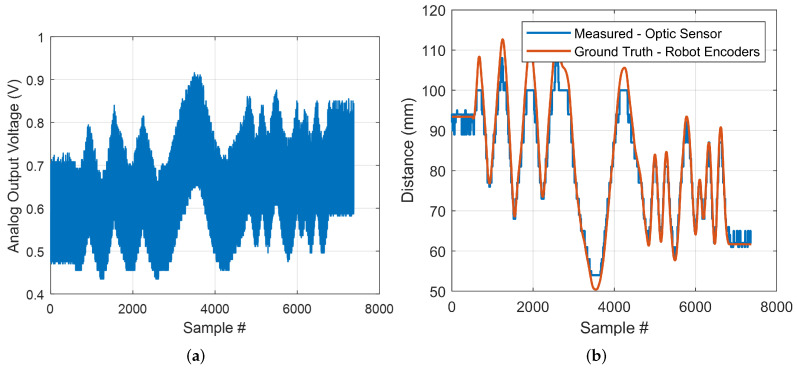
Sample of data collected for distance measurement accuracy evaluation (**a**) sensor’s recorded raw output voltage; (**b**) distance measured by the sensor and the reference values.

Figure 4 shows a sample of data collected for the evaluation of measurement accuracy. The robot tip moved in a random sinusoidal pattern on the surface of the tissue sample. Figure 4a shows the raw voltage recorded. Figure 4b presents the distance measured by the sensor using Equation (7) and the reference value based on the known sensor position attached to the robot’s tip. In this experiment, the RMS error for the silicone tissue sample was 3.6 mm. According to the results, the sensor measured distances more accurately between 54 mm and 100 mm, which is within the manufacturer’s reported working range.

In the next step, the coordinate frame transformation between the sensor frame, end-effector frame, and robot base frame was used to calculate the 3D point cloud of the tissue surface sample points. At each time step, measured distances $d_{tip}$ were associated with robot joints data. The measured distances represent the location of the tissue sample point in the frame attached to the sensor, as shown in Figure 1. The coordinates of each sampled point on the tissue surface expressed in the robot base frame ${}^{B}$$P_{cl}$ were calculated using the following equation based on the orientation and position of the sensor frame at each time step:

(8) $${}^{B}P_{cl}={}^{B}T_{E}{}^{E}T_{S}{}^{S}P_{cl}$$

where ${}^{S}P_{cl}={\left[0,0,d_{tip},0\right]}^{T}$ is the position of the sampled point on the tissue surface expressed in the sensor frame, ${}^{E}T_{S}$ is the transformation matrix from sensor frame to end-effector frame, and ${}^{B}T_{E}$ is the transformation matrix from the end-effector frame to a robot base frame calculated using a forward kinematic model of the robot.

**Figure 5:**
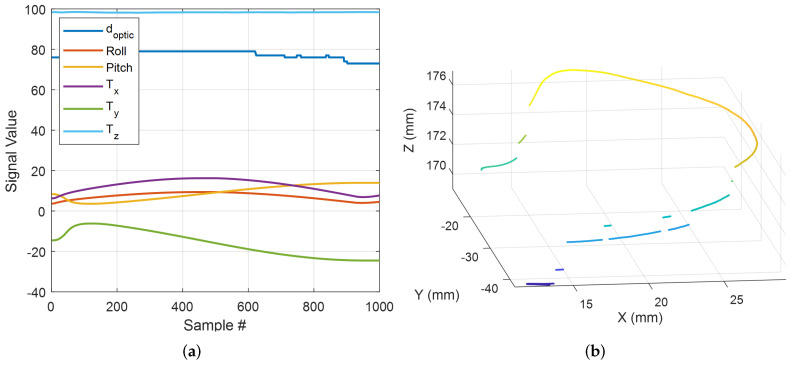
3D point cloud generation (**a**) a sample of tissue surface scanning procedure raw data; (**b**) 3D point cloud of the tissue surface generated from the raw dataset.

Figure 5a shows an example of raw data recorded during the scanning of a tissue surface, including the end-effector tip coordinates $\left[T_{x},T_{y},T_{z}\right]$, the robot’s roll and pitch DOF, and the optical sensor measured distances $d_{tip}$. The 3D point cloud of the tissue surface generated from this dataset using Equation (8) is shown in Figure 5b. A discontinuity in the path of the point cloud was caused by the optical sensor’s inaccuracy, as shown in this figure. This issue was overcome in the next step using a robust plan estimation algorithm.

Estimation of tissue surface was performed using the maximum likelihood estimator sample consensus algorithm (MLESAC) in Matlab. This algorithm is a modified and robust version of the common random sample consensus (RANSAC) algorithm [52]. The MLESAC algorithm separated the data into two groups of inliers and outliers and then dismissed the outliers so that it could predict the data based only on the inliers. Plane solutions were chosen that maximize the likelihood in the presence of outliers instead of only considering data with a higher number of inliers. Next, the algorithm calculated the error term and modeled it as a mixture of Gaussian and uniform distributions. A negative log-likelihood was then calculated to minimize the error.

Using recorded 3D point clouds of the tissue surface, the algorithm was tested for accuracy. The experiment was repeated ten times for four known plane orientations with 0°, 5°, 10°, and 20° tilt. The RMS of the estimation error for each of the planes from 0° to 20° were 1.8°, 2.1°, 3.2°, and 4.7°, respectively. The plane estimation error was higher for larger plane angles due to limitations in sensor measurement since sensor accuracy greatly impacts plane estimation results.

### 4.2. Hyperplanar Virtual Fixture Impedance Control

The impedance control strategy was established to control the dynamic impedance of the haptic device and adopt the VF guidance forces. Impedance dynamics equation for a phantom haptic device interacting with the user in Cartesian space model is as follows:

(9) $$M_{m}\ddot{\tilde{x}}+C_{m}\dot{\tilde{x}}+G_{m}=f_{h}+f_{m}$$

where $M_{m}\in R^{3\times 3}$ is the positive definite inertia matrix, $C_{m}\in R^{3\times 3}$ is the damping matrix, $G_{m}\in R^{3}$ is the gravitational force vector, $\tilde{x}=x_{d}-x$ is the difference between the desired value for the position vector $x_{d}$ in the task space, and the actual position vector $x\in R^{3}$. $f_{h}\in R^{3}$ is the human applied force on the master device, and $f_{m}\in R^{3}$ is the vector of master tool control forces that are generated for the implemented VF force $f_{vf}$ and the transmitted sensed tool–environment forces $f_{e}$.

Due to the lack of a force sensor on the master tool, the user-applied force vector $f_{h}$ cannot be directly measured. As a result, the following force estimator was implemented:

(10) $${\hat{f}}_{h}=M_{m}\ddot{\tilde{x}}+C_{m}\dot{\tilde{x}}+G_{m}-f_{m}$$

Estimated force was assumed to equal the actual applied force $\left(f_{h}={\hat{f}}_{h}\right)$. Calculation of master device control forces $f_{m}$ for VF force feedback was then carried out.

**Figure 6:**
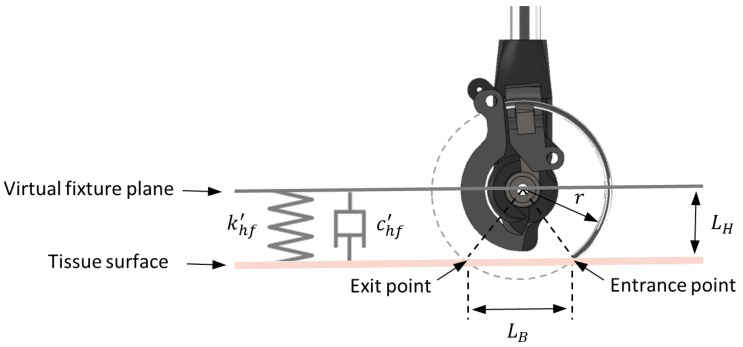
Illustration of the hyperplanar VF spring–damper forces for maintaining the desired $L_{H}$ distance between the needle centre of rotation and tissue surface plane for achieving the selected bite length $L_{B}$.

Figure 6 illustrates the hyperplanar VF spring–damper forces that guide the user toward a desired plane. Using the geometrical relationships, it can be shown that the desired bite length $L_{B}$ is associated with the distance $L_{H}$ between the needle centre of rotation and the tissue surface plane as follows:

(11) $$L_{H}=r\;\cos\;\left(\sin^{-1}\;\left(L_{B}/2r\right)\right)$$

In this equation, *r* represents the radius of the needle curve, and $L_{B}$ represents the distance between the entrance and exit points of the needle.

The hyperplanar VF constrains the slave robot tip to move within a plane and maintains the distance $L_{H}$ calculated by Equation (11). The tissue plane can be defined based on the identified orientation in Section 4.1, with the unit normal vector $\hat{n}=\left(n_{x},n_{y},n_{z}\right)$ and the user selected point ${}^{B}P_{S}$. The following parallel constraint plane was defined above the tissue plane:

(12) $$\begin{array}{cc} P_{tissue}:n_{x}x+n_{y}y+n_{z}z+d & =0 \end{array}$$

(13) $$\begin{array}{cc} P_{VF}:n_{x}x+n_{y}y+n_{z}z+d+L_{H} & =0 \end{array}$$

The closest point ${}^{B}x_{r}^{cl}$ on the constraint plane $P_{VF}$ and the robot end-effector centre of rotation ${}^{B}x_{r}$ can be calculated using the projection of the point ${}^{B}x_{r}$ on the $P_{VF}$ plane as follows:

(14) $${}^{B}x_{r}^{cl}={}^{B}P_{S}^{cl}-\;\left(\left({}^{B}x_{r}-\;{}^{B}P_{S}^{cl}\right)\cdot \hat{n}\right)\;\hat{n}$$

where ${}^{B}P_{S}^{cl}$ is the projection of the user selected point ${}^{B}P_{S}$ on the $P_{VF}$ plane.

The desired master tool position, $x_{md}$, at each time step, can be calculated using the mapping equation between the desired salve robot position ${}^{B}x_{r}^{cl}$ on the constraint plane $P_{VF}$ as follows:

(15) $${}^{M}x_{md}=\alpha_{P}^{-1}{}^{M}R_{B}{}^{B}x_{r}^{cl}$$

A spring–damper guidance force was modeled to maintain the end-effector centre of rotation within the constraint plane:

(16) $$f_{m}=f_{hf}=K_{hf}\;{\tilde{x}}_{m}+C_{hf}\;{\dot{\tilde{x}}}_{m}$$

where ${\tilde{x}}_{m}=x_{md}-x_{m}$ is the difference between the desired and actual master tool position. $K_{hf}$ and $C_{hf}$ are the hyperplanar VF spring and damper coefficient diagonal matrices, respectively.

Subsequently, the haptic force felt by the user can be calculated based on Equation (10) as follows:

(17) $${\hat{f}}_{h}=M_{m}\;{\ddot{\tilde{x}}}_{m}+\left(C_{m}-C_{hf}\right)\;{\dot{\tilde{x}}}_{m}-K_{hf}\;{\tilde{x}}_{m}+G_{m}$$

$f_{h}$ force guided the user toward the closest point on the hyperplane. The direction of $f_{h}$ changed based on the position of the end-effector on either side of the plane, bringing the user back to the desired distance above the tissue.

## 5. Experimental Research Facility

**Figure 7:**
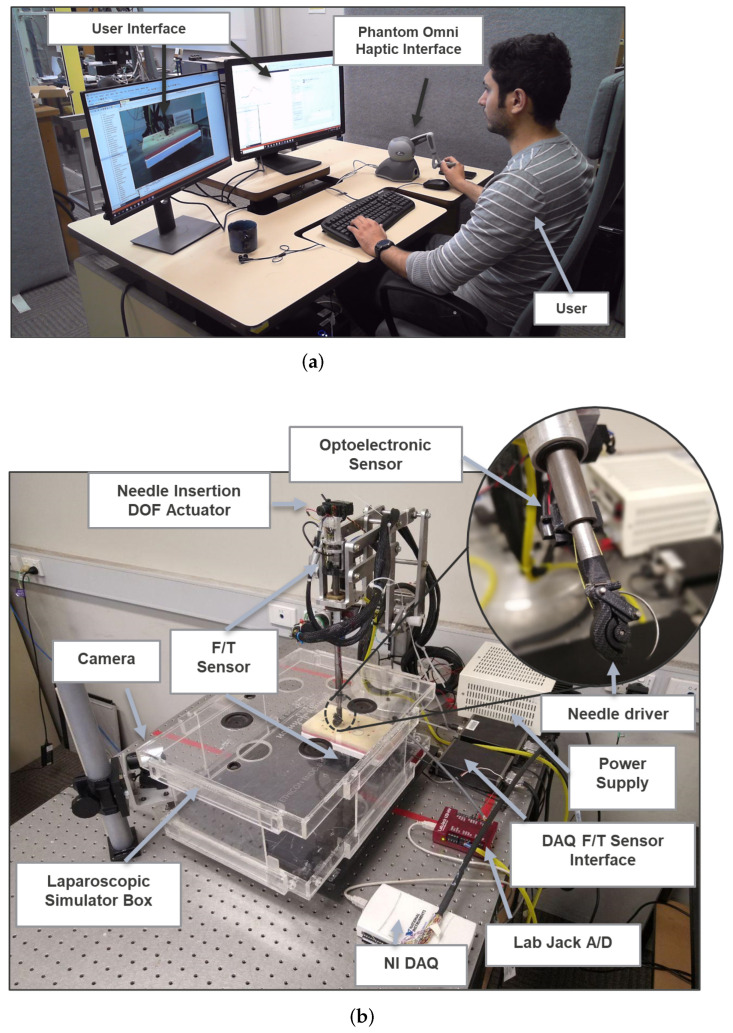
Experimental research facility: (**a**) user interface and haptic device; (**b**) teleoperated robotic surgical system platform.

The experimental research facility for this study is shown in Figure 7. The user interacted with the robotic system using a C++ command interface connected to Matlab via a Matlab Engine API and a Phantom Omni haptic device. C++ and Matlab were both processed on the same PC and communicated via TCP/IP. Teleoperated robotic surgical systems were originally developed in [53,54]. Commands for slave robot were transmitted to Maxon EPOS 2 motor controllers using the CAN-bus protocol.

The infrared optoelectronic sensor used for distance measurement was Sharp IR distance sensor GP2Y0A51SK0F, designed to measure distances of 2–15 cm. Voltage data were sampled at a rate of 60 Hz due to the sensor’s limitations in providing higher sampling rates. Datasheets indicated that the optical sensor could detect distances from surfaces with as little as 0.18 reflective grey paper. In the data synchronization process, the interpolation technique was used to calculate synchronized values for data collected at different rates to match the robot 200 Hz control loop.

Haptic feedback was provided by a Phantom Omni with 6-DOF position sensing and 3-DOF force feedback. The Phantom Omni standard IEEE-1394 FireWire communication protocol was used for user interaction with the haptic device. Motion commands were taken from the user by manipulating the stylus in different positions and orientations and transferring them to the robot control loop. Through the actuation of the joints in 3 Cartesian coordinates, feedback force data were transmitted to the user. The maximum force that the actuators could produce was 3.3 N, and the position sensing resolution was 0.055 mm.

In this study, Ethicon J351H curved needles with 40 mm needle length and 29 mm needle diameter were used with the scaled fabricated end-effector. A Johnson and Johnson laparoscopic simulator box was used for the experiments. In order to measure needle–tissue interaction forces, a silicone surgical training pad was installed on top of an ATI Mini40 force-torque sensor (ATI Industrial Automation). Force data were transmitted at 30 kHz and smoothed with a 300 sample moving average filter, resulting in an effective 100 Hz rate.

## 6. Results

### 6.1. Hyperplanar Virtual Fixture

The user performed a series of needle insertion trials with each trial involving stitching into eight evenly spaced needle entrance points on a circle of 40 mm diameter in two different modes. The needle driver was manually loaded with a new needle between needle insertion cycles. As part of the first mode, the user was provided with hyperplanar VF forces to maintain the end-effector centre of rotation at a fixed $L_{H}$ distance above the tissue using VF geometry determined by the optoelectronic sensor. A second mode allowed the user to move the end-effector in any direction without being guided. The user repeated this task for ten trials in each mode. The tissue plane was placed horizontally, and hence the only VF force that the user received was normal to the tissue surface to keep the tool above the tissue within the VF plane.

**Figure 8:**
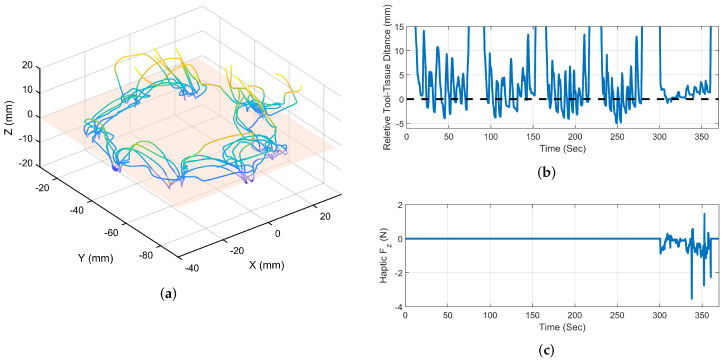
Needle insertion task results for no hyperplanar VF assistance mode. (**a**) 3D path of the needle tip in space toward the desired entrance point; (**b**) relative position of the end-effector centre of rotation above the VF plane; (**c**) user received force $F_{h}$.

Figure 8a shows a sample of suturing trails data of the end-effector’s trajectory in the second mode and identified VF hyperplane that indicates the ideal location for the end-effector. Figure 8b demonstrates the relative distance between the end-effector and VF hyperplane. Ideally, the end-effector should be located within the VF plane, as this produces the desired bite length. Therefore, the relative distance between the end-effector and VF plane should be as small as possible. To avoid contact with the tissue surface, the needle tip had to be moved away from the tissue surface. As a result, the user needed to adjust the $L_{H}$ distance repeatedly. For each marked needle entrance point, a $L_{H}$ distance adjustment was made individually. A number of accidental contacts were also observed when the end-effector passed through the VF plane, shown as negative relative positions. In the fifth trial, the results of VF-activated mode and receiver guidance force are shown to compare modes and demonstrate how effective VF assistance is at reducing repeated tool height re-alignments. Based on this data, it is evident that the algorithm maintains the $L_{H}$ distance and reduces the operator’s workload for adjusting end-effector positions.

**Figure 9:**
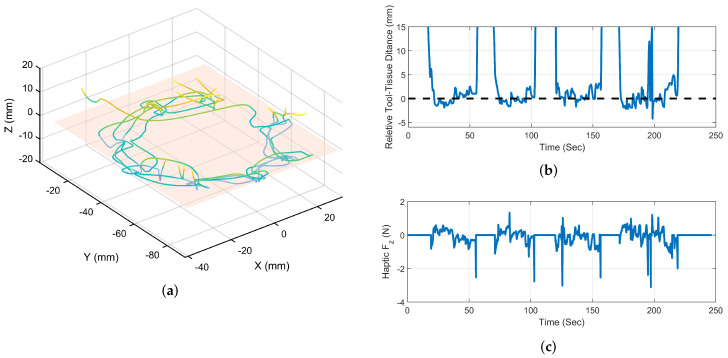
Needle insertion task results for hyperplanar VF assistance mode. (**a**) 3D path of the needle tip in space toward the desired entrance point; (**b**) relative position of the end-effector centre of rotation above the VF plane; (**c**) user received force $F_{h}$.

Figure 9a illustrates a sample result of the end-effector’s trajectory during VF-assisted trials. Figure 9b shows the relative distance between the end-effector’s centre of rotation and the VF plane, as well as the user forces $f_{h}$. Due to the horizontal VF plane, the user only received the $F_{z}$ force component normal to the tissue surface. In response to the user’s movements above the tissue plane, the $F_{z}$ sign changes to match the position of the end-effector relative to the VF plane. A change in $F_{z}$ direction was observed as the tool was moved out of the VF plane from its two sides. As a result, the user was able to maintain the end-effector movement within the VF plane with minimal fluctuations above the tissue plane with the assistance of the VF forces.

During the needle insertion task trials, the average time for the first and second modes was 36.4 s and 54.2 s, respectively. The average needle entrance point error for the second mode was 2.4 mm, while the average for the VF-assisted mode was 1.5 mm.

### 6.2. Data-Based Interaction Force Estimation and Haptic Feedback Implementation

**Figure 10:**
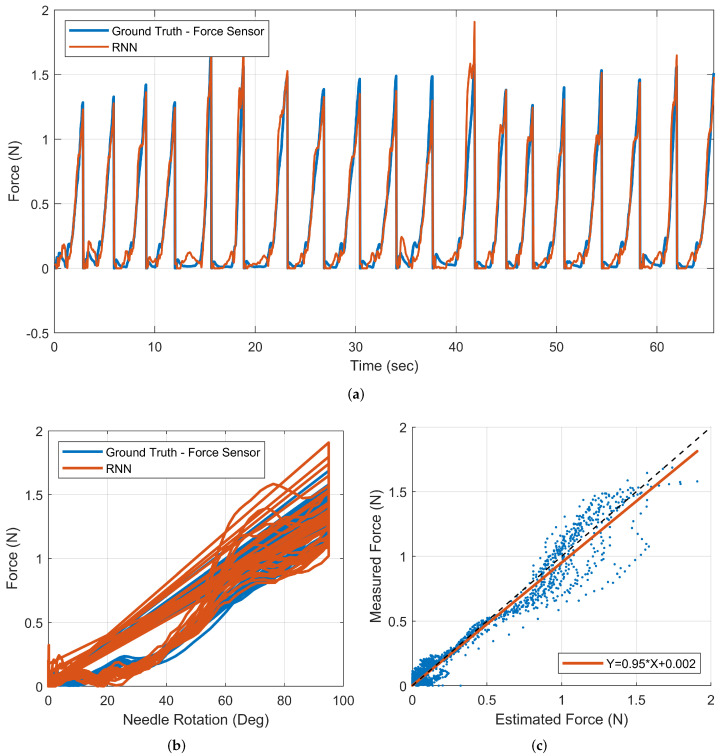
Results for a sample output of the RNN model. (**a**) the force–time profile of the recorded force versus the estimated force calculated by the force model; (**b**) the force–needle rotation profile; (**c**) fitted line to the pairs of the target values versus the estimated force values with an ideal slope of 1 shown with a black dashed line.

The selected RNN model was tested with real-time and unseen data samples. The force estimation results are presented in Figure 10. Figure 10a displays the actual and estimated results in a time series. Using this model, the estimation error was −0.01 N, with a standard deviation of 0.09 N. In addition, the RMSE for the test sample was calculated to be 0.15 N. Figure 10b shows the model’s ability to follow the force–needle rotation profile. A first-order line was fitted using a least squares approach to the pairs of the target values and estimated force to determine the overall accuracy of the force model. It is ideal for the slope of target–output pairs to be equal to one. Figure 10c shows the fitted line to the selected model target–output pairs. The dashed black line indicates the ideal slope for this curve fit. A slope of 0.95 was calculated for the RNN network. According to this result, the RNN model performed well in modelling the needle insertion process with a 5% error from the ideal slope line.

As part of the last experiment, the user received direct feedback forces in two modes: VF-assisted and without VF. The needle insertion actuator was activated by holding down a button on the haptic device stylus and moving the stylus along the *x*-axis of the master device coordinate frame. The haptic device provided the user with the online needle–tissue interaction force estimations calculated from Equation (6). The transferred forces were smoothed using a low pass filter applied to ${\hat{f}}_{hx}$. In addition, the user was presented with a visual graph displaying real-time estimated forces.

**Figure 11:**
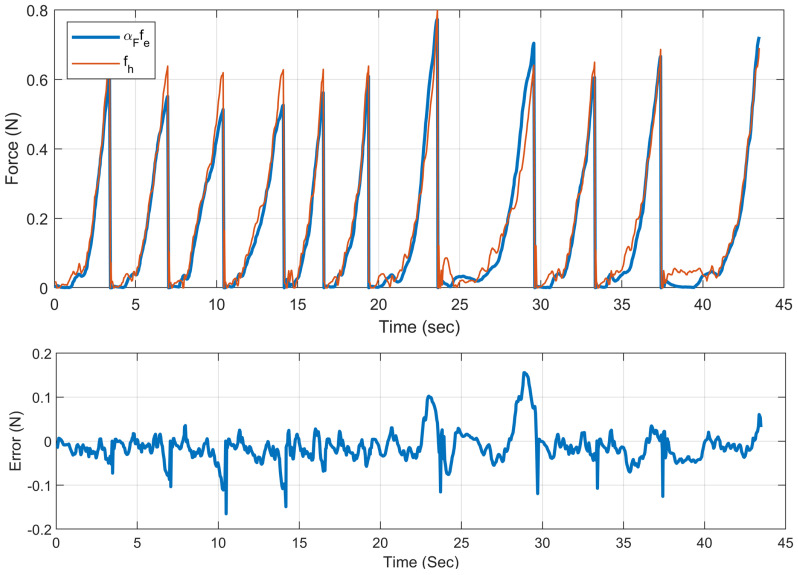
Force–time profile of the scaled actual needle–tissue interaction forces recorded by the F/T sensor under the tissue versus the user received force.

Figure 11 shows a force–time profile of the needle–tissue interaction forces from the F/T sensor under the tissue, as well as the haptic force provided to the user. The perceived user force $f_{h}$ is shown in red, and the scaled ground truth value ($\alpha_{F}f_{e}$) is shown in blue. Within the first 20 s, interaction data are shown in VF-assisted hyperplanar mode, and from 20–45 s, the non-VF mode is presented. There was a 0.07 N RMSE between user-perceived forces and the scaled environment forces in the sample results. According to the graph, the interaction forces were relatively higher in the non-VF assistance mode due to inaccurate $L_{H}$ adjustments. Specifically, this is caused by excessive forces created by the end-effector pushing the needle tip down on the surface of the tissue when the ideal $L_{H}$ distance is not maintained.

## 7. Discussion and Conclusions

This paper presented a teleoperated suturing methodology using a force-sensing needle driver that semi-automates the MIS stitching task and provides an indirect measurement of needle–tissue contact forces.

Initially, an indirect force-sensing method was developed. Using indirect force measurement models, robot sensors were mapped to needle–tissue forces. The interaction force was estimated using a data-based force estimation model. In order to test the accuracy of force estimation models, online input data from the slave robot motor kinematics and the instrument force sensor were used, and the results were compared with the ground truth force profile that was acquired from tissue-integrated force sensors. As a result of the simplified end-effector mechanism, indirect force estimation was possible with high accuracy, thus facilitating intuitive force feedback. Additionally, these results demonstrated the potential of data-based force estimation for force feedback. Using the force-sensing instrument developed, bilateral teleoperation robotic methodology was established. An impedance control scheme was used to cancel the dynamics of the master device and to transmit scaled estimations of the environmental forces from needle–tissue interaction to the user. Experimental evaluation and verification of the proposed robotic stitching methodology were conducted. The haptic feedback experimental results indicated the ability of the proposed teleoperated robotic needle driver to convey the interaction force data with 0.07 N RSME between the actual scaled environment and user perceived forces.

A reflective optoelectronic sensor-based approach for estimating the tissue plane orientation was investigated, using the scanned 3D point cloud of the tissue surface and the MLESAC algorithm results. A hyperplanar virtual fixture was constructed based on the estimated orientation of the tissue. The VF algorithm was established via impedance control of the master haptic device. Compared to laser-based scanning or depth cameras, a reflective optic sensor’s data analysis computational costs are significantly lower. There is a potential for reducing the duration of real-time data processing with this benefit. In addition, the sensor is considerably less expensive than an image-based measurement camera. This reduces the system’s overall cost and even makes it suitable for disposable instruments. Experimental results showed that the proposed VF was capable of reducing robot manipulation time, increasing task accuracy, and reducing interaction forces. The hyperplanar VF also restricted the robot manipulation to a plane with the desired distance above the identified tissue surface, which resulted in the desired bite length. In conclusion, the proposed methodology can potentially decrease the complexity of the tool’s operation, ease the burden on the surgeons, and improve the accuracy and repeatability of the task.

A number of extensions can be made to this research to improve the needle driver’s capability. Investigating a needle driver mechanism that retrieves the needle after needle insertion could significantly improve the suturing process. This feature requires removing passive clamping provided by mechanical stoppers and developing active clamping capability for the jaws that grasp the needle tip using the same degree of freedom. Furthermore, the proposed methodologies can be extended to other surgical skills such as dissection and retraction, which can greatly benefit from the force-feedback capabilities of the system. It is possible to improve the force-sensing by investigating data-based models with three outputs that could estimate each force component separately. It is also likely that cable friction can affect estimation accuracy, requiring future research to incorporate additional pulleys into the instrument. The force-sensing capability of the instrument in the presence of tissue deformation can be investigated. The force models evaluated were developed based on the assumption of minimal tissue deformation utilizing the experimental data from a tissue installed on a firm base. In future studies, the accuracy of data-based models can be improved with training data sets collected from experiments, including vertical and horizontal tissue deformations. Finally, further research will assess how the proposed instrument compares with the traditional instrument using objective scoring tools, such as robotic assessment and competency evaluation (RACE).

## Data Availability

Not applicable.
